# Cesáreas como expresión biopolítica del nacimiento: el caso de la provincia del Neuquén, Argentina

**DOI:** 10.18294/sc.2026.6032

**Published:** 2026-05-15

**Authors:** Serena Perner, Hugo Spinelli, Viviana Martinovich, Marcio Alazraqui

**Affiliations:** 1 Doctora en Salud Colectiva. Docente-Investigadora, Instituto de Salud Colectiva, Universidad Nacional de Lanús, Buenos Aires, Argentina. serenaperner@gmail.com Universidad Nacional de Lanús Instituto de Salud Colectiva Universidad Nacional de Lanús Buenos Aires Argentina serenaperner@gmail.com; 2 Doctor en Salud Colectiva. Investigador, Instituto de Salud Colectiva, Universidad Nacional de Lanús, Buenos Aires, Argentina. hugospinelli09@gmail.com Universidad Nacional de Lanús Instituto de Salud Colectiva Universidad Nacional de Lanús Buenos Aires Argentina hugospinelli09@gmail.com; 3 Doctora en Salud Colectiva. Docente-Investigadora, Instituto de Salud Colectiva, Universidad Nacional de Lanús, Buenos Aires, Argentina. vivianamartinovich@gmail.com Universidad Nacional de Lanús Instituto de Salud Colectiva Universidad Nacional de Lanús Buenos Aires Argentina vivianamartinovich@gmail.com; 4 Doctor en Salud Colectiva. Director, Instituto de Salud Colectiva, Universidad Nacional de Lanús, Buenos Aires, Argentina. malazraqui@yahoo.com.ar Universidad Nacional de Lanús Instituto de Salud Colectiva Universidad Nacional de Lanús Buenos Aires Argentina malazraqui@yahoo.com.ar

**Keywords:** Nacimiento, Cesárea, Biopolítica, Argentina, Birth, Cesarean Section, Biopolitics, Argentina

## Abstract

Las cesáreas aumentan a nivel global a pesar de las recomendaciones internacionales y de la evidencia sobre sus riesgos en los casos que no están clínicamente justificadas. Este estudio caracteriza la tendencia temporal de indicadores vinculados a los nacimientos vivos en la provincia del Neuquén, Argentina, y la analiza desde la biopolítica del nacimiento, que entiende la cesárea, no solo como técnica quirúrgica, sino como un dispositivo complejo, una tecnología biopolítica que actúa simultáneamente sobre múltiples planos. Se realizó un análisis de tendencias temporales a partir de fuentes de información provinciales, reconstruyendo indicadores de natalidad, mortalidad infantil y tipo de parto desde 1970 hasta 2022. Para el período entre 2011 y 2022 se estudió la relación de los cambios en el tiempo en la tasa de cesáreas según variables socioeconómicas y asistenciales, mediante modelos lineales generalizados. Los resultados muestran un incremento sostenido de las cesáreas -del 20% en 1995 al 50% en 2022- en un contexto de descenso de la natalidad y de la mortalidad infantil, con patrones diferenciales según nivel educativo, edad de la persona gestante, tipo de establecimiento y organización temporal de los nacimientos vivos. Estos hallazgos permiten interpretar la expansión de las cesáreas como parte de un proceso más amplio de regulación y normalización de las formas de nacer, en los que la cesárea se configura como una tecnología que contribuye a producir previsibilidad y establecer estándares sobre cuándo y cómo debe ocurrir el nacimiento, creando subjetividades que reconfiguran las condiciones sociales y simbólicas del nacimiento.

## Abstract (en)

Cesarean sections continue to increase globally regardless of international recommendations and evidence of their risks when not medically indicated. This study characterizes the time-trend of birth indicators in the province of Neuquén, Argentina, through the perspective of the biopolitics of birth, which understands the cesarean section not merely as a surgical technique, but as a complex device, a biopolitical technology that acts simultaneously on multiple levels. A time-trend analysis was conducted using provincial administrative records, reconstructing indicators of birth rate, infant mortality, and delivery mode from 1970 to 2022. For the period 2011 and 2022, the relationship between changes over time in the cesarean section rate according to socioeconomic and healthcare variables was studied using generalized linear models. The results show a sustained increase in cesarean sections - from 20% in 1995 to 50% in 2022 - in a context marked by declining birth rates and infant mortality, along with patterns that vary according to educational level, age of the pregnant people, type of healthcare facility, and timing of births. These findings suggest that the rise in cesarean sections can be interpreted as part of a broader process of regulating and standardizing modes of giving birth, in which the cesarean section is configured as a technology that contributes to producing predictability and establish standards regarding when and how birth should occur, thereby shaping subjectivities that reshape the social and symbolic conditions of birth.

## Introducción

Según la Organización Mundial de la Salud, las cesáreas continúan aumentando en el mundo^1^^,^^2^, a la par de un incremento del número de nacimientos prematuros inmaduros con un mayor riesgo de padecer diferentes enfermedades crónicas a lo largo de la vida^3^^,^^4^^,^^5^^,^^6^^,^^7^^,^^8^^,^^9^. En 2021, a nivel global, los partos por cesárea correspondían a más de 1 de cada 5 partos (21%) con una situación heterogénea entre regiones y países. Según proyecciones, esa cifra seguirá en aumento y, para la década de 2030 se estima que llegará casi a un tercio (29%) del total de partos^1^^,^^2^. Este fenómeno no es reciente^10^^,^^11^, diferentes estudios en países de América Latina y el Caribe vienen señalando el incremento de las cesáreas desde la década de 1970^12^^,^^13^^,^^14^^,^^15^^,^^16^^,^^17^.

A pesar de las recomendaciones internacionales de una tasa de cesáreas de alrededor del 15%, estas siguen aumentando en el mundo^1^^,^^2^. Si bien es una intervención médica eficaz para salvar vidas cuando está clínicamente justificada, su uso sin justificación incrementa los riesgos de complicaciones tanto en las personas gestantes, como de quienes nacen por cesárea^18^^,^^19^^,^^20^. Entendemos que este incremento responde a que la cesárea, más que una técnica quirúrgica, es un dispositivo complejo, una tecnología política que pone en acción una *biopolítica del nacimiento* que actúa simultáneamente sobre múltiples planos.

### Biopolítica del nacimiento

Desde la perspectiva de Michel Foucault, el disciplinamiento del cuerpo y la regulación vital de la población constituyen “los dos polos alrededor de los cuales se desarrolló la organización del poder sobre la vida”^21^, de allí que la *biopolítica* reduce las experiencias vitales al dominio de lo calculable, de lo controlable y “convierte al poder-saber en un agente de transformación de la vida humana”^21^. Así, las *tecnologías biopolíticas* pasan a “invadir el cuerpo, la salud, las maneras de alimentarse y alojarse, las condiciones de vida, el espacio entero de la existencia”^21^. De este modo, la biopolítica desplaza el control sobre el cuerpo individual hacia la gestión de los procesos vitales de la población^22^, como la reproducción, la natalidad, la mortalidad o la morbilidad. El nacimiento deja de ser un acontecimiento singular y se convierte en un objeto de saberes e intervenciones, susceptibles de ser normalizados. En ese marco, las cesáreas funcionan no solo como una intervención clínica, sino como una tecnología biopolítica que estructura sentidos, prácticas, tiempos y decisiones en torno al nacimiento.

Por un lado, la biopolítica transforma el parto en un proceso anticipable y programable, que sustituye la temporalidad incierta del trabajo de parto por una temporalidad administrativa compatible con las agendas de las y los profesionales de la salud, y con los turnos, protocolos e intereses del amplio espectro de instituciones del campo de la salud. El nacimiento se integra así a la racionalidad organizacional de la institución médica. Pero, por otro lado, en esa expansión de las cesáreas participan también la producción de una subjetividad compartida por el conjunto social. 

En términos de Marcuse^23^, la institucionalización del dominio técnico sobre las acciones sociales logra un nivel de aceptación y de legitimidad tal que las decisiones quedan atravesadas por la subordinación (no solo de las personas gestantes, sino de la sociedad en su conjunto) a un aparato técnico que, a la vez que instala la supresión del dolor como horizonte normativo de la experiencia vital, aumenta la productividad en el campo de la salud. De este modo, el discurso del “parto riesgoso”, del “sufrimiento evitable” o del “bienestar fetal” no se imponen, sino que se internalizan, interpelando a las personas gestantes como responsables morales del resultado biológico del nacimiento. Así, el cuerpo gestante es construido como un cuerpo potencialmente fallido, peligroso o imprevisible, que requiere vigilancia constante^24^, lo que se potencia aún más en los cuerpos de las personas gestantes con discapacidad^25^. La cesárea se transforma así en una respuesta tecnificada que reduce la imprevisibilidad y el dolor propio de la experiencia vital del parto, aspectos que parecerían alcanzar tal legitimidad, que incluso penetran en entornos basados en la humanización de la atención^26^.

En esa interfaz entre tecnificación de la vida planteada por Marcuse y la biopolítica foucoultiana, Nikolas Rose señala que “la vitalidad humana […] se ha vuelto terreno franco para la innovación técnica, la explotación económica y formas de bioeconomía fuertemente competitivas. Esta tecnologización y capitalización de la medicina otorgan una forma singular al disputado campo de la política vital en el siglo XXI”^27^. 

Aun así, esa política vital o biopolítica del nacimiento no actúa de manera homogénea. Las cesáreas revelan desigualdades de clase^16^, género, territorio en el campo de la salud, mostrando que no todos los nacimientos son gobernados del mismo modo^28^. En algunos contextos, la cesárea es sobreutilizada; en otros, es inaccesible; y, en otros contextos, se van resquebrajando los acuerdos respecto al parto humanizado y las cosmovisiones propias de los pueblos originarios respecto del parto, de tal manera que tanto la cosmovisión de estas otras culturas queda relegadas ante el poder institucionalizante de la tecnificación del parto y la biopolítica del nacimiento^29^. 

### El caso de la provincia del Neuquén

Neuquén es una provincia ubicada en el noroeste de la región patagónica argentina, región de baja densidad poblacional y que, según los datos del censo 2022^30^, presenta la mayor población que se autopercibe como mapuche exclusivamente. Es la primera provincia argentina en tener un establecimiento sanitario intercultural^31^, el Centro de Salud Intercultural Raguiñ Kien, emplazado en la Cuenca Ruca Choroi. Inaugurado en 2021, pero como resultado de años discusiones y espacios de consenso, forma parte del sistema público de salud de la provincia, con un modelo de atención basado en el abordaje intercultural, en el que se conjuga la biomedicina con la medicina mapuche^32^^,^^33^^,^^34^, en un marco complejo de encuentro de saberes^34^.

En la década de 1950, la provincia del Neuquén adquiere estatus político provincial y crea un fuerte sistema público de salud, basada en centros de salud de alcance territorial, médicas y médicos rurales y agentes sanitarios^35^, y un sistema hospitalario de complejidad creciente, aún vigente^36^^,^^37^. Esa apuesta implicó un compromiso social y una firmeza política, que permitió construir un sistema de salud que ha sido ejemplo no solo para otras provincias, sino para otros países de América Latina^34^. A partir de la invitación a radicarse en la provincia a sanitaristas y profesionales de la salud, comenzó a plasmarse en Neuquén el programa de salud rural, con agentes sanitarios que, desde entonces, enfrentaron diferentes desafíos para poder llegar a las poblaciones rurales. El sistema siguió un modelo que centralizó la administración y diversificó sus alcances para optimizar las respuestas desde los diferentes efectores sanitarios^38^^,^^39^^,^^40^. 

En 1970, la implementación del “Plan de salud”, tuvo como principal objetivo llevar la atención sanitaria a la población de todo el territorio, en contraposición a modelos que focalizaban la atención en hospitales centrales. Así, la provincia del Neuquén definió un horizonte sanitario basado en la equidad y en la universalidad, con una atención integral de salud y prácticas centradas en la maternidad e infancia, logrando vincular al gobierno provincial directamente con la comunidad a través de acciones de promoción, prevención, atención y rehabilitación de la salud, poniendo especial énfasis en el nivel de atención/cuidado, favoreciendo la descentralización geográfica y dando prioridad a las necesidades sociales, al abordar problemáticas de salud desde una perspectiva familiar y territorial. Ese primer nivel de atención resultó fundamental en la prevención de enfermedades, en la promoción de la salud y en una más eficiente relación de la atención de la salud con los niveles de mayor complejidad, caracterizado por ofrecer asistencia en el territorio a cada paciente que la requiriera antes de decidirse su internación. De esta forma, se configuró una red de atención de complejidad creciente, en la que el hospital no fue la primera instancia de atención, garantizando así la eficiencia en el tratamiento y la atención más adecuada de cada paciente^35^^,^^36^^,^^39^.

Hasta la década de 1970, gran cantidad de los nacimientos en la provincia del Neuquén eran domiciliarios, atendidos por parteras empíricas y machis (figura central de la medicina mapuche). Paulatinamente, las instituciones públicas comenzaron a institucionalizar el parto en el hospital, considerado como uno de los logros del Plan de Salud^40^. Esto produjo que entre 1970 y 2006 los partos domiciliarios descendieran del 27% al 0,2%^36^^,^^41^. En el marco de ese proceso, a partir de la década de 1990, en la provincia comienza un crecimiento de las instituciones privadas y, en ese contexto, se produce un progresivo aumento de las cesáreas, desde la primera cesárea realizada en la provincia en 1957, a un 20% de nacimientos por cesárea en 1995 (primer año del que se encontró registro oficial del dato), al 50% en 2022. Frente al aumento del parto por cesárea, en 2021, el Ministerio de Salud de la provincia del Neuquén generó un protocolo para garantizar el derecho a un parto respetado^42^, en el que se explicita, como primer punto, el derecho “a un parto respetuoso de los tiempos biológicos y psicológicos, evitando prácticas invasivas y suministro de medicación que no esté justificada”^42^. Aun así, las cesáreas siguen aumentando. 

Desde este contexto, nos proponemos caracterizar la tendencia temporal de diferentes indicadores vinculados a los nacimientos vivos en la provincia de Neuquén y analizarlos no solo desde su dimensión epidemiológica, histórica y asistencial, sino entendiendo la cesárea como un dispositivo complejo que pone en acción una *biopolítica del nacimiento* que estructura sentidos y prácticas, tiempos y decisiones en torno al nacimiento.

## Metodología

La posibilidad de cuantificar estos eventos se vincula a la calidad de las estadísticas sanitarias. En Argentina no hay datos oficiales que permitan calcular la tasa de cesárea, ya que no es un aspecto sistemáticamente relevado en el *Informe estadístico de Nacido Vivo* publicado por el Ministerio de Salud de la Nación. Existen datos parciales del subsector público a partir del Sistema Informático Perinatal (SIP), herramienta de la OPS empleada por el Ministerio de Salud de Argentina, o datos de determinadas instituciones privadas^43^^,^^44^. 

La provincia del Neuquén, en particular, se caracteriza por la buena calidad de sus registros y, desde la década de 1970^37^, presenta un sistema de recolección continua de información referente a nacidas/os vivas/os y también de las defunciones. Se trata de una de las pocas jurisdicciones de Argentina que disponibilizó la forma de terminación del parto en su sistema de información en salud, lo que permite analizar el cambio en el tiempo de las cesáreas. 

Por un lado, se realizó un análisis temporal a partir de fuentes de información provinciales, en las que se reconstruyeron indicadores de natalidad, mortalidad infantil y el tipo de parto desde 1970 hasta 2022. Para esta construcción se relevaron las siguientes fuentes oficiales y publicaciones: el Plan Quinquenal 1984-1988 del Ministerio de Salud Pública de Neuquén^45^, la página web de la Dirección Provincial de Estadística y Censos de la provincia del Neuquén^46^ y publicaciones con informes provinciales^42^^,^^47^. En estas fuentes se buscó la siguiente información para el total provincial: cantidad de nacimientos vivos (NV) por año, cantidad de población por año, mortalidad infantil y tipo de parto. Esto permitió calcular los siguientes indicadores para cada año y elaborar la tendencia temporal: tasa de natalidad (nacimientos vivos por 1.000 habitantes), tasa de mortalidad infantil (muertes de menores de un año de edad por 1.000 NV) y porcentaje de cesáreas (nacimientos vivos por cesárea por 100 NV). Se calculó la variación proporcional porcentual (VPP) de las series, entre 1970 y 2022, mediante el siguiente cálculo ((tasa final-tasa inicial)/tasa inicial) x 100.

Por otro lado, a partir de información individual de cada nacimiento disponible en los registros de nacimientos de 2011 a 2022 de la Dirección Provincial de Estadística y Censos se pudo analizar la relación de los cambios en el tiempo según el tipo de parto (cesáreas o parto vaginal) relacionándolo con variables socioeconómicas y asistenciales. En total, se incluyeron los 119.097 registros de nacimientos vivos de personas gestantes en ese período, quienes, al momento del nacimiento, vivían en la provincia de Neuquén, Argentina. 

Como variables socioeconómicas se incluyeron el nivel educativo de la persona gestante (bajo: con secundaria incompleta o menos; medio: con secundaria completa o universitario incompleta; y alto: universitaria completa o más) y la edad de la persona gestante (19 años o menos, 20 a 34 años, 35 años y más). Como variables asistenciales, el lugar del parto (establecimiento público o privado) y variables relativas al tiempo (año, día de la semana y hora del parto). 

Para este segundo análisis, se analizó primero la frecuencia a lo largo del tiempo (en tres cuatrienios: 2011-2014, 2015-2018 y 2019-2022) de nacimientos vivos y de las cesáreas según las distintas variables analizadas. Luego se analizó la distribución de los partos por cesárea y partos vaginales según días de la semana y según la hora del nacimiento, comparando, en ambos casos, según tipo de establecimiento (público y privado). 

Luego se estimó la tasa de cesáreas al inicio del periodo y su cambio en el tiempo según categorías de nivel educativo, de edad y del tipo de establecimiento. Para esto se usaron modelos lineales generalizados, familia binomial. Se calculó la razón de prevalencia con el intervalo de confianza del 95% (IC95%), ya que el cálculo de los odds ratio (OR), tiende a sobreestimar la asociación para un evento frecuente como la cesárea. Finalmente, se realizó una regresión de Poisson para predecir los cambios en el tiempo de las tasas de cesáreas según nivel educativo (usando como numerador el recuento de cesáreas y como denominador el total de nacimientos). Todos los análisis se realizaron el STATA® 18. 

Se respetó la Ley de Secreto estadístico que protege la confidencialidad de la información contenida en las estadísticas vitales (Informe Estadístico de Nacido Vivo).

## Resultados

### Análisis de indicadores vinculados a los nacimientos vivos entre 1970-2022

**Figura 1:**
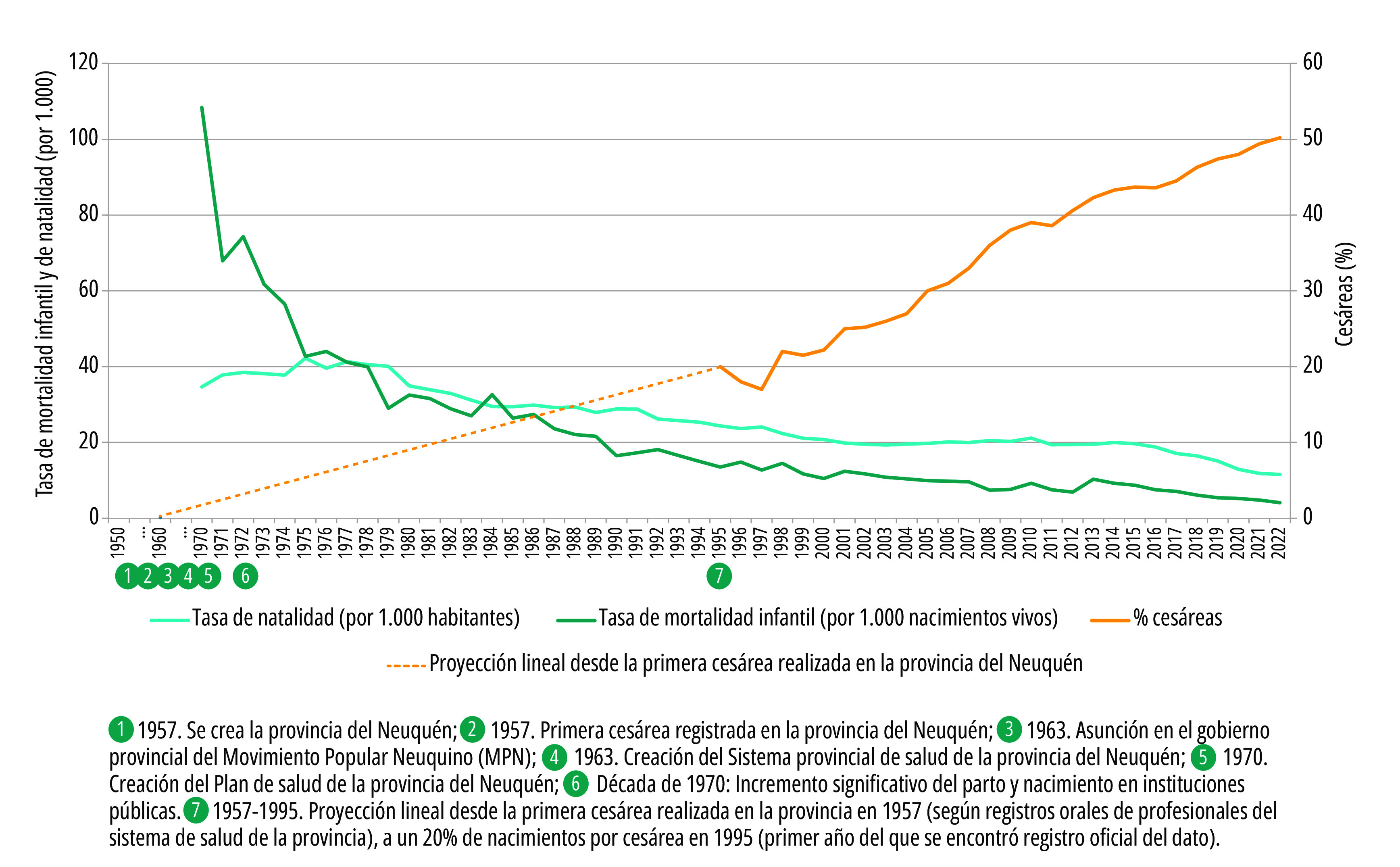
Tendencia temporal de indicadores epidemiológicos vinculados a la salud materno-infantil, con referencias a eventos sociosanitarios y políticos relevantes. Provincia del Neuquén, Argentina, 1950-2022.

Fue posible reconstruir la tendencia de natalidad y de mortalidad infantil, desde el año 1970 hasta 2022, y de cesáreas, desde el año 1995. Estos indicadores fueron graficados en la Figura 1, en la que se incluyen, además, diferentes eventos históricos sociosanitarios relevantes para la provincia desde mediados del siglo XX. La información sobre los dos primeros indicadores comienza unos pocos años luego de la creación del Sistema Provincial de Salud, en 1963, y de la creación del Plan de Salud de la provincia, en 1970. 

Fuente: Elaboración propia con base en Dirección General de Información y Control^45^; Consejo Federal de Inversiones *et al*.^48^; Dirección Provincial de Estadística, Censos y Documentación^49^^,^^51^; Dirección Provincial de Estadística y Censos ^50^.

Destacamos el pronunciado descenso que se observa en los primeros 10 años (hasta 1980) en la tasa de mortalidad infantil (de 108,4 por 1.000 NV en 1970 a 32,5 por 1.000 NV en 1980, VPP: -70,1%). Luego de 1980, continua con un descenso ininterrumpido en el tiempo hasta alcanzar una tasa de 4,1 por 1.000 NV en 2022. La tasa de natalidad también disminuyó a lo largo del tiempo, pasando de 34,7 NV por 1.000 habitantes en 1970 a 11,5 NV por 1.000 habitantes en 2022 (VPP: -66,8%). Desde comienzos del nuevo siglo hasta el año 2014, la tasa de natalidad se mantuvo entre 19,3 y 20,5 NV por 1.000 habitantes, y desde allí comenzó un descenso año tras año, llegando a la mitad de esos nacimientos en 2022 (11,5 NV por 1.000 habitantes). 

Según registros aportados por profesionales del sistema de salud de la provincia, la primera cesárea en Neuquén data de 1957; y, para 1995 -año en que comienza el registro oficial y sistemático del indicador-, las cesáreas ya representaban el 20% de los nacimientos, y de allí en más ascendió de manera constante hasta llegar al 50% en 2022, mostrando un fuerte entrecruzamiento en las tendencias de los indicadores (Figura 1). 

Siguiendo con el análisis temporal, en términos absolutos, la población neuquina creció de manera sostenida a lo largo de todo el periodo (de 154.570 habitantes en 1970 a 680.726 en 2022). Por su parte, la cantidad de nacimientos vivos aumentó hasta 2010 (pasando de 5.360 NV en 1970 a 12.100 NV en 2010), momento a partir del cual comenzó a descender hasta situarse, en 2022, en valores similares a los de la década de 1970 (7.859 NV en 2022). 

### Análisis de las cesáreas entre 2011-2022

Además, a partir de 2011, cuando se comenzó a obtener información de variables individuales, se observaron otros cambios a lo largo del tiempo en el patrón de nacimientos vivos: un cambio en la edad de las mujeres al momento del nacimiento (descenso de la proporción de nacimientos de mujeres menores de 19 años y un ascenso de aquellas con 35 años y más), y un descenso en la proporción de nacimientos entre las mujeres de menor nivel educativo. En cuanto al tipo de parto, observamos un incremento en la proporción de cesáreas en el período 2011-2022 (del 38% al 50%) y un incremento de los nacimientos los días hábiles respecto a aquellos ocurridos en fines de semana o feriados (Tabla 1). 

**Tabla 1:** Distribución de nacimientos vivos, según características socioeconómicas y tipo de parto (vaginal o cesárea). Neuquén, Argentina, 2011-2022.

Características socioeconómicas	2011 a 2014		2015 a 2018		2019 a 2022		Total	
	n= 44.545		n= 42.629		n=31.923		n=119.097	
	%	n	%	n	%	n	%	n
**Edad de la persona gestante**								
19 años o menos	15,6	6.930	12,5	5.319	7,9	2.521	12,4	14.770
20 - 34 años	70,3	31.313	71,0	30.247	73,1	23.351	71,3	84.911
35 y más años	14,1	6.301	16,6	7.062	19,0	6.050	16,3	19.413
Sin dato	0,0	1	0,0	1	0,0	1	0,0	3
**Nivel educativo de la persona gestante^1^**								
Bajo	41,8	18.645	37,3	15.918	39,0	12.442	39,5	47.005
Medio	32,5	14.485	31,4	13.386	41,3	13.173	32,8	39.044
Alto	12,2	5.426	14,4	6.146	19,3	6.152	14,9	17.724
Sin dato	13,4	5.989	16,8	7.177	0,5	156	12,9	15.324
**Tipo de parto**								
Vaginal	59,1	26.332	56,0	23.851	51,6	16.482	56,0	66.665
Cesárea	40,9	18.213	44,0	18.778	48,4	15.433	44,0	52.424
Sin dato	-	0	-	0	0,1	8	0,0	8
**Lugar del parto**								
Institución pública	55,1	24.535	53,9	22.967	55,8	17.820	54,8	65.322
Institución privada	44,7	16.930	45,9	19.568	44,0	14.047	45,0	53.545
Otro lugar^2^	0,1	8	0,1	10	0,0	3	0,1	21
Vivienda particular	0,1	64	0,2	74	0,2	51	0,2	189
Sin dato	0,0	8	0,1	10	0,1	2	0,1	20
**Días de la semana**								
Días hábiles	76,6	34.100	80,9	34.485	80,3	25.629	79,1	94.214
Sábados, domingos, feriados	23,4	10.445	19,1	8.144	19,7	6.294	20,9	24.883

Fuente: Elaboración propia con base en estadísticas vitales de la provincia del Neuquén. ^1^Nivel educativo de la persona gestante: baja (hasta secundaria incompleta); media (secundaria completa o universitario, terciario incompleto); alto (universitaria o terciario completo). ^2^Vía pública, transporte, etc.

**Figura 2:**
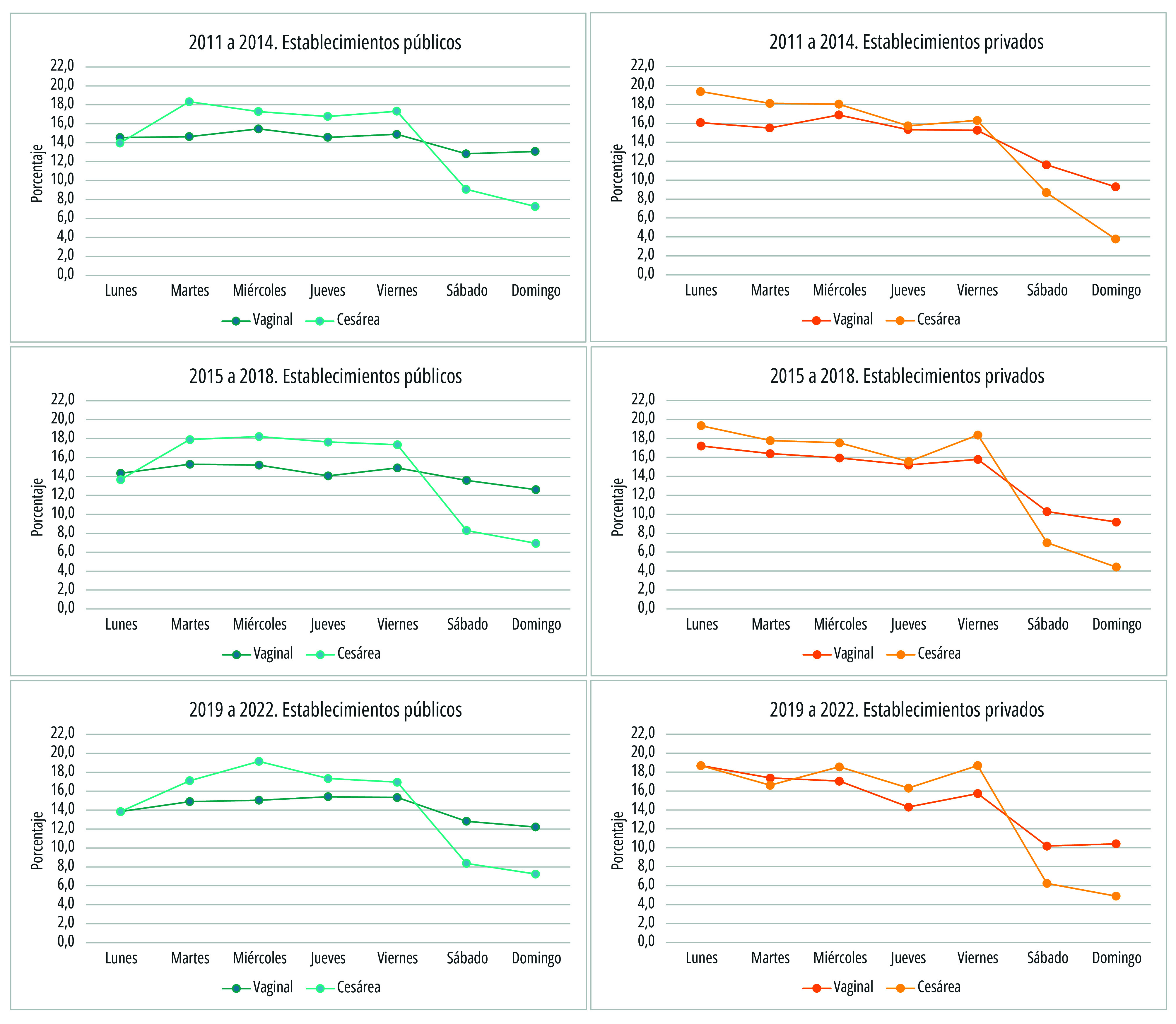
Porcentaje de nacimientos vivos por día de la semana, según tipo de parto (vaginal o cesária) y tipo de establecimiento (público o privado). Neuquén, Argentina, 2011-2022.

Los nacimientos vivos presentaron diferencias entre establecimientos con financiamiento público o privado en su distribución temporal, relacionada con los horarios de trabajo. Entre los nacimientos en establecimientos públicos, los partos vaginales se mantuvieron relativamente estables a lo largo de los días de la semana, pero las cesáreas se concentraron de lunes a viernes, lo que se mantuvo estable a lo largo del tiempo (distintos cuatrienios). Sin embargo, en los establecimientos privados, tanto los partos vaginales como las cesáreas se concentraron principalmente de lunes a viernes en los tres cuatrienios (Figura 2). 

Fuente: Elaboración propia con base en estadísticas vitales de la provincia del Neuquén.

**Figura 3:**
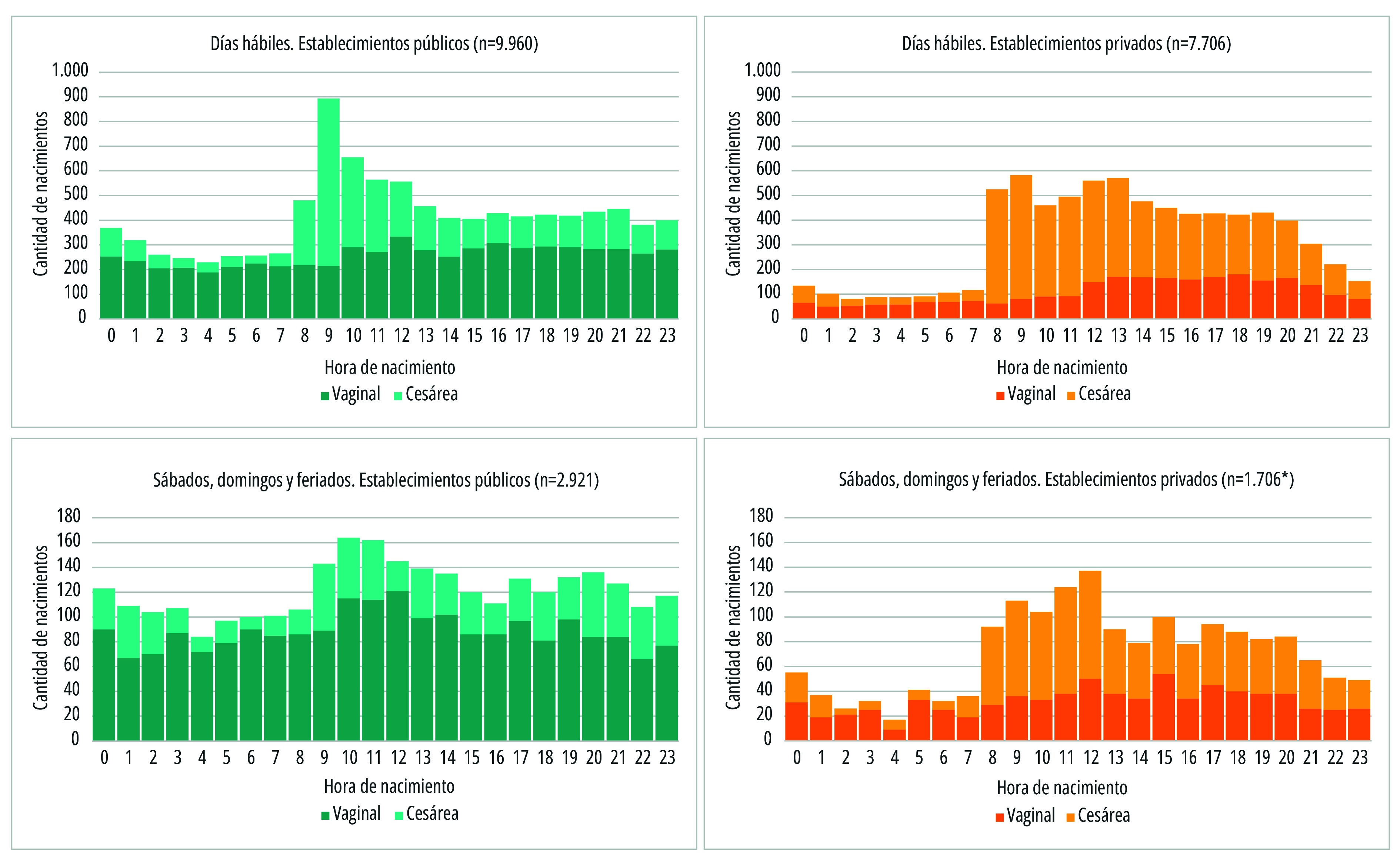
Número de nacimientos vivos por hora y día de la semana, según tipo de parto (vaginal o cesárea), y tipo de establecimiento (público o privado). Neuquén, Argentina, 2019-2022.

En la Figura 3 se grafica la distribución de los nacimientos vivos por hora y tipo de parto, estratificado por establecimientos públicos y privados, según días hábiles o sábados, domingos y feriados. Entre los establecimientos privados, todos los días de la semana los nacimientos se concentran a partir de las 8:00 de la mañana hasta las 20:00 hs y luego descienden hasta llegar a valores bajos en horas de la noche. En particular, los nacimientos por cesárea se concentran a partir de las 12:00 hs los días hábiles, en cambio los sábados, domingos y feriados no se observa gran cambio a lo largo del día. En los nacimientos en establecimientos públicos, se observa un cambio en el patrón de los nacimientos vivos: de lunes a viernes se observa una mayor proporción de nacimientos por la mañana y luego valores menores desde las 14:00 hasta la 1:00 de la madrugada, y un leve descenso entre las 2:00 y las 7:00 de la mañana. Los sábados, domingos y feriados se ve un patrón más estable a lo largo del día, en el que la proporción de cesáreas es más estable. ¿Estas variaciones responden al parto como un acontecimiento humano singular o a su transformación en un objeto de intervención anticipable y programable?

Fuente: Elaboración propia con base en estadísticas vitales de la provincia del Neuquén. Nota: Se tomó solo el tercer cuatrienio ya que 2019 es el primer año con información sobre la hora de nacimiento en establecimientos privados. Datos faltantes para estos 4 años: privado = 33% (12.881/17.677), público = 27% (9.412/13.990)

El análisis descriptivo de las variables presentadas en la Tabla 2 muestra un incremento en la proporción de cesáreas a medida que aumenta la edad de las mujeres, tanto entre los nacimientos en establecimientos públicos como en privados. Con relación al nivel educativo, observamos diferencias solo entre los nacimientos en el sistema público (a mayor nivel educativo, más cesáreas) no así en el privado, donde se diluyen esas diferencias. Finalmente, observamos un incremento de las cesáreas a lo largo del tiempo en ambos tipos de establecimientos. 

**Tabla 2:** Proporción de cesáreas según características socioeconómicas, por tipo de establecimiento (público o privado). Neuquén, Argentina, 2011-2022.

Características socioeconómicas	Nacimientos vivos en establecimientos públicos			Nacimientos vivos en establecimientos privados			Total de nacimientos vivos		
	Nacimientos vivos	Cesáreas		Nacimientos vivos	Cesáreas		Nacimientos vivos	Cesáreas	
	n	n	%	n	n	%	n	n	%
Total	65.315	19.817	30,4	53.544	32.597	61,0	119.089	52.424	44,0
**Edad de la mujer**									
19 años o menos	10.602	2.489	23,5	4.144	2.271	54,8	14.769	4.762	32,2
20 - 34 años	46.693	14.342	30,7	38.051	22.635	59,5	84.904	36.983	43,6
35 y más años	8.019	2.986	37,2	11.348	7.691	67,8	19.413	10.678	55,0
**Nivel educativo de la mujer**									
Bajo	35.241	10.011	28,4	11.680	6.960	59,6	47.456	16.964	35,7
Medio &nbsp;	17.956	5.994	33,4	21.024	13.002	61,8	39.041	19.000	48,7
Alto	4.186	1.579	37,7	13.506	8.236	61,0	17.723	9.817	55,4
**Cuatrienios (período)**									
2011-2014	24.535	7.025	28,6	19.930	11.186	56,1	44.545	18.213	40,9
2015-2018	22.967	6.718	29,3	19.568	12.054	61,6	42.629	18.778	44,1
2019-2022	17.813	6.075	34,1	14.046	9.357	66,6	31.915	15.433	48,9
**Días**									
Días hábiles	48.904	16.406	33,5	44.367	28.272	63,7	93.438	44.687	47,8
Sábados, domingos, feriados	15.723	3.412	21,7	8.898	4.325	48,6	24.679	7.737	31,4

Fuente: Elaboración propia con base en estadísticas vitales de la provincia del Neuquén. Nota: Nivel educativo: Bajo (primaria completa o menos); Medio (secundaria completa o universitaria incompleta); Alto (universitaria completa o más).

En la Tabla 3 se muestran las tendencias en las tasas de cesáreas según las variables socioeconómicas seleccionadas. En total, las cesáreas se incrementan en un 11% cada 5 años [RP=1,10; IC95% (1,10-1,12)]. Respecto a la edad de las personas gestantes, si bien al inicio de la tendencia la tasa fue mayor entre las personas gestantes de 35 años y más, el incremento fue mayor entre las personas gestantes más jóvenes, de 19 años o menos. Respecto al nivel educativo, al inicio del período, las tasas fueron más elevadas entre aquellas personas gestantes de mayor educativo; sin embargo, el incremento cada 5 años en la tasa entre aquellas personas de nivel educativo más bajo fue del 10% [RP=1,10; IC95% (1,08-1,12)] y 5% entre las de nivel educativo más alto [RP=1,05; IC95% (1,03-1,07)]. Finalmente, en relación con el tipo de establecimiento sanitario, al inicio de la tendencia, las tasas fueron mayores entre aquellos nacimientos en establecimientos privados, y se observó un incremento del 11% cada 5 años entre aquellos nacimientos en establecimientos públicos [RP=1,11; IC95% (1,09-1,13)] y 9% entre los nacimientos en establecimientos privados [RP=1,09; IC95% (1,08-1,10)].

**Tabla 3:** Tendencia temporal de cesáreas, según características socioeconómicas. Neuquén, Argentina, 2011-2022.

Características socioeconómicas	Valores al inicio de la tendencia (2011)		Tendencia temporal	
	RP^a^	IC95%	RP^a^	IC95%
**Tiempo**				
Cada 5 años	-	-	1,11	1,10-1,12
**Edad de la persona gestante**				
19 años o menos	1,00	-	1,10	1,06-1,15
20 a 34 años	1,33	1,27-1,39	1,10	1,09-1,11
35 y más años	1,72	1,64-1,80	1,07	1,05-1,09
**Educación de la persona gestante**				
Baja	1,00	-	1,10	1,08-1,12
Media	1,32	1,28-1,36	1,07	1,06-1,09
Alta	1,48	1,43-1,53	1,05	1,03-1,07
**Tipo de establecimiento**				
Público	1,00	-	1,11	1,09-1,13
Privado	1,94	1,89-2,00	1,09	1,08-1,10

Fuente: Elaboración propia con base en estadísticas vitales de la provincia del Neuquén. RP: razón de prevalencias. IC95%: intervalo de confianza del 95%. Nivel educativo: Bajo (Primaria completa o menos); Medio (Secundaria completa o universitaria incompleta); Alto (Universitaria completa o más) ^a^Valores crudos sin ajustar. Estimación mediante modelos lineales generalizados, familia binomial.

**Figura 4:**
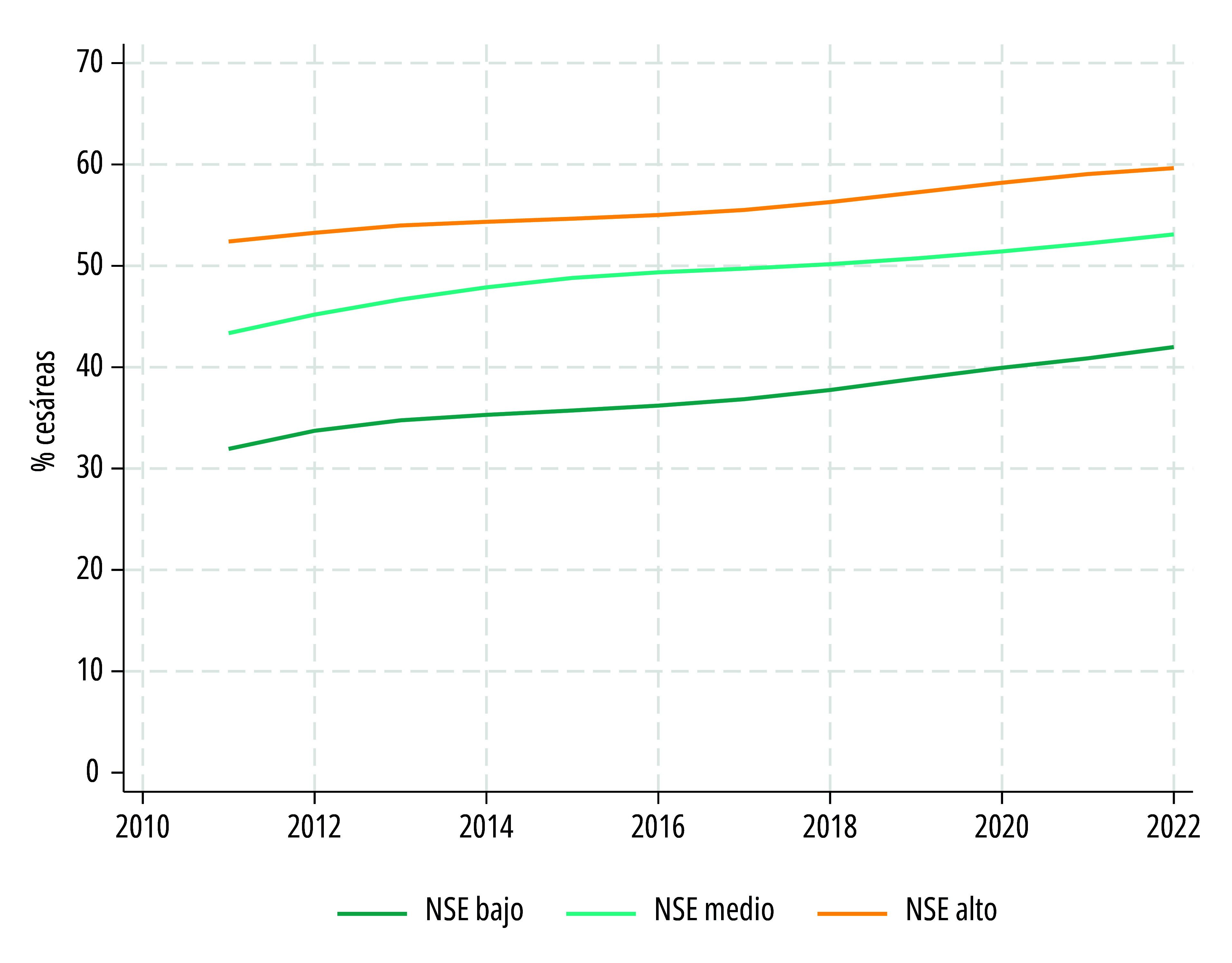
Tendencia estimada del porcentaje de cesáreas, según nivel socioeconómico de la persona gestante. Neuquén, Argentina, 2011-2022.

En la Figura 4 se observan los cambios en el tiempo en las tasas de cesáreas según nivel socioeconómico. Resaltamos que, en todo el período, la tasa de cesáreas fue superior entre las mujeres de mayor nivel educativo. Sin embargo, el incremento en el tiempo fue más acentuado entre las mujeres de nivel educativo bajo (incremento en 10 años del 12,3% entre las de nivel educativo alto, 16,9% en el nivel medio y del 23,8% entre las de nivel bajo). 

Fuente: Elaboración propia con base en estadísticas vitales de la provincia del Neuquén. NSE= Nivel socioeconómico. NSE bajo= Hasta secundaria incompleta. NSE medio= secundaria completa o universitaria, terciario incompleto. NSE alto= universitaria, terciario completo

## Discusión

En el caso de Argentina, este análisis fue posible en la provincia del Neuquén, dado que la recolección de estadísticas a fin de monitorear las políticas sanitarias fue parte de los ejes del plan de salud instaurado en la provincia en la década de 1970, estrategia que se mantuvo a lo largo de las décadas siguientes. 

A partir del análisis de las series históricas y de los registros individuales de nacimientos en la provincia de Neuquén, se identifica un incremento sostenido de las cesáreas en el tiempo, que pasan del 20% en 1995 al 50% en 2022, proceso se da en un contexto de privatización, que lleva a un aumento de las instituciones privadas en la provincia de Neuquén, en detrimento de las instituciones públicas. 

El incremento identificado de las cesáreas coincide con las tendencias globales y regionales de 154 países que muestran un aumento de las tasas de cesáreas en todas las regiones desde 1990. Según el estudio de Betran *et al.*^10^*,* la región con las tasas de cesáreas más altas es América Latina y el Caribe (42,8%), región en la que se encuentran los dos países con la tasa más alta de cesáreas en todo el mundo: República Dominicana (58,1%) y Brasil (55,7%).

Mientras indicadores clásicos de salud materno-infantil, como la natalidad y la mortalidad infantil, muestran un descenso sostenido desde la década de 1970, la tasa de cesáreas se contrapone a esta tendencia, mostrando un cambio en las prácticas del parto, aspecto que se acentúa al observar la distribución temporal (en horas y días de la semana) de los nacimientos. Por el contrario, estos resultados sugieren que el crecimiento de las cesáreas se explicaría por transformaciones en las formas de organización, regulación e intervención sobre el nacimiento, más que por un incremento de los eventos que requieren atención obstétrica.

La tendencia al uso excesivo e innecesario de las cesáreas es motivo de gran preocupación a nivel mundial, dados los riesgos que entraña para las personas gestantes y para el recién nacido^52^. Entre esos riesgos se han reportado complicaciones maternas evitables, como infecciones, hemorragias, complicaciones relacionadas con el uso de anestesia o transfusiones de sangre, rotura uterina, problemas de implantación placentaria y necesidad de histerectomía^20^^,^^53^^,^^54^ y en la niñez, problemas respiratorios, dermatitis atópica y obesidad^55^^,^^56^^,^^57^^,^^58^. 

A pesar de estos riesgos, las estimaciones muestran que la cesárea ya se ha convertido en la modalidad de parto más frecuente en varios países^10^, lo que revela que, más allá de factores y procesos específicos de cada país, la cesárea opera como un dispositivo complejo que actúa simultáneamente sobre múltiples planos que regulan, gestionan y normalizan las formas de nacer a nivel global. Desde la biopolítica del nacimiento, la cesárea se consolida como una tecnología orientada a gestionar la incertidumbre del parto, producir previsibilidad en un proceso biológico intrínsecamente incierto, mercantilizando y tecnificando el mundo vital. De hecho, las altas tasas de cesáreas también están asociadas con un aumento considerable de los costos sanitarios, lo que implica una carga para los sistemas de salud^55^. En términos biopolíticos, el nacimiento deja de ser un acontecimiento singular para convertirse en un proceso regulado, más predecible, controlable y fácil de supervisar^59^, en el que la cesárea programada se transforma en una solución para la gestión del tiempo^60^^,^^61^.

Por otro lado, los patrones temporales de los nacimientos con concentración en días hábiles y horarios diurnos son coincidentes con trabajos anteriores^16^^,^^62^^,^^63^, y muestran una organización del nacimiento ajustada a lógicas laborales e institucionales, y exponen de manera empírica cómo la cesárea opera como una tecnología que alinea los tiempos biológicos con los tiempos institucionales, integrando el nacimiento a la racionalidad organizacional de los establecimientos de salud, pero también a las demandas (laborales y subjetivas) de las personas gestantes^63^^,^^64^. 

En contraposición a una mirada que concibe a las personas gestantes como actores pasivos de sus propios partos^65^, entendemos que la biopolítica del nacimiento atraviesa a todo el cuerpo social. En *Microfísica del poder*, Foucault señala que las relaciones de poder pueden “penetrar materialmente en el espesor mismo de los cuerpos sin que la representación de los sujetos tenga que relevarlas”^66^. La expansión de la cesárea participa así de relaciones de poder y de la producción de una subjetividad social compartida, en la que la cesárea no se impone, sino que construye un escenario donde elegirla aparece como una decisión racional, técnicamente neutra y moralmente correcta.

En términos de Foucault, la biopolítica opera un poder-saber que transforma la vida humana^21^. Así, el poder-saber de la medicina pone en acción mecanismos de transformación del parto y administración del cuerpo que envuelven a otros agentes, como los proveedores sanitarios, que reciben incentivos para emplear tecnologías sanitarias que proporcionan un monitoreo e intervención constante, lo que da lugar a la medicalización del embarazo y el parto^16^^,^^67^ impulsada, además, por la mercantilización de la salud y por los intereses económicos sobre la salud^68^. Esas articulaciones le permiten a la racionalidad médica relegar a un segundo plano la dimensión cuidadora de la persona gestante y el niño por nacer^69^, y producir una experiencia tecnificada y medicalizada del parto que, en algunos casos, es interiorizada y exigida por las propias personas gestantes^70^ y, en otros, es atravesada como una experiencia indeseada, que fragmenta el cuerpo y los deseos de un parto humanizado^71^. 

Otro de los hallazgos es que las tasas más elevadas corresponden a personas gestantes de nivel educativo alto, lo que muestra cómo la cesárea se asocia históricamente a determinados capitales sociales y culturales, como también el uso del tiempo, el miedo al dolor, la reproducción de normas sociales^67^ o la percepción de calidad asistencial^72^; mientras que el avance progresivo en los niveles educativos bajos sugiere que se va ampliando su alcance y normalización en distintos grupos sociales. 

El incremento de las cesáreas en Neuquén, con tasas históricamente más elevadas en el subsector privado, pero con un incremento proporcionalmente mayor en el subsector público en los últimos años, indica que no se trata de una práctica restringida a un modelo de financiamiento específico, sino de una lógica transversal de gobierno del nacimiento, que atraviesa diferentes regímenes institucionales. La mayor aceleración en el sector público en los últimos años sugiere, además, una creciente convergencia de racionalidades asistenciales, que ha sido identificada también en Brasil, en el marco del Sistema Único de Salud^24^^,^^26^^,^^73^^,^^74^.

Finalmente, el aumento de las cesáreas entre personas gestantes más jóvenes y la persistencia de tasas elevadas aun en contextos con políticas explícitas de parto respetado y humanización de la atención^26^ refuerzan la idea de que la biopolítica del nacimiento no opera principalmente por imposición, sino por producción de consentimiento y de subjetividades. 

En conjunto, los resultados analizados permiten sostener que la cesárea, lejos de ser únicamente una respuesta clínica a situaciones de riesgo, funciona como una tecnología biopolítica que no solo no elimina el riesgo, sino que lo produce como categoría de intervención. En este sentido, López *et al*. señalan que, incluso en el marco del Sistema Único de Salud (SUS) de Brasil, donde la humanización ha sido definida como una perspectiva orientadora de las políticas públicas brasileñas, “el parto por cesárea es construido como la alternativa más ‘segura’ frente a los ‘riesgos’ del parto vaginal”^73^. 

Desde esta perspectiva, la cesárea aparece como una tecnología central de la biopolítica del nacimiento, al permitir traducir la variabilidad del parto en procedimientos compatibles con las lógicas organizacionales del campo de la salud, aun en contextos donde existen políticas explícitas de promoción del parto respetado, como es el caso de la provincia del Neuquén.

Como limitaciones, podemos resaltar, entre otras, la falta de datos sobre etnia en los registros de nacimientos vivos, situación que se repite en otros registros oficiales de nuestro país, silenciando e invisibilizando diferencias étnicas que puedan dar cuenta de brechas de acceso y desigualdades sanitarias. 

## Conclusiones

Los resultados muestran que en los 27 años que transcurrieron entre 1995 y 2022, la tasa de cesáreas en Neuquén tuvo un incremento relativo del 150% respecto al valor inicial, en paralelo con un marcado descenso de la natalidad y de la mortalidad infantil. El crecimiento de las cesáreas parece vincularse con transformaciones más profundas en las prácticas asistenciales y en las formas de organización institucional del nacimiento, que exceden el plano estrictamente clínico. 

El análisis muestra, además, que las cesáreas se distribuyen de manera diferencial según variables sociodemográficas e institucionales, con mayores prevalencias entre personas gestantes de mayor nivel educativo, y en edades maternas más avanzadas. Asimismo, la concentración de cesáreas en días hábiles y horarios diurnos evidencia que el nacimiento se organiza según lógicas institucionales y laborales que tienden a producir previsibilidad en un proceso biológico caracterizado por su incertidumbre. 

Desde la biopolítica del nacimiento, los resultados permiten interpretar la expansión de las cesáreas como parte de un proceso más amplio de regulación y normalización de las formas de nacer. En este sentido, la cesárea se configura como una tecnología que contribuye a producir previsibilidad y establecer estándares implícitos sobre cuándo y cómo debe ocurrir el nacimiento. Más que una respuesta exclusivamente clínica a situaciones de riesgo obstétrico, la cesárea aparece como un dispositivo que articula dimensiones médicas, organizacionales, sociales y simbólicas. 

Finalmente, este estudio pone en evidencia que la articulación entre análisis epidemiológico y reflexión teórica abre una agenda orientada a comprender fenómenos sociales complejos, que exceden el plano estrictamente clínico, y abre caminos para comprender cómo los procesos poblacionales de salud se configuran en la intersección entre dinámicas biomédicas, racionalidades institucionales, intereses económicos y transformaciones sociales más amplias.
